# Bioactive Secondary Metabolites from *Trichoderma* spp. against Phytopathogenic Bacteria and Root-Knot Nematode

**DOI:** 10.3390/microorganisms8030401

**Published:** 2020-03-13

**Authors:** Raja Asad Ali Khan, Saba Najeeb, Zhenchuan Mao, Jian Ling, Yuhong Yang, Yan Li, Bingyan Xie

**Affiliations:** Institute of Vegetables and Flowers, Chinese Academy of Agricultural Sciences, Beijing 100081, China; asadraja@aup.edu.pk (R.A.A.K.); sabanajeeb831@gmail.com (S.N.); maozhenchuan@caas.cn (Z.M.); lingjian2005@126.com (J.L.); yangyuhong@caas.cn (Y.Y.)

**Keywords:** secondary metabolites, *Trichoderma* spp., phytopathogenic bacteria, root-knot nematode

## Abstract

Losses in crops caused by plant pathogenic bacteria and parasitic nematode are increasing because of a decrease in efficacy of traditional management measures. There is an urgent need to develop nonchemical and ecofriendly based management to control plant diseases. A potential approach of controlling plant disease in the crops is the use of biocontrol agents and their secondary metabolites (SMs). Luckily fungi and especially the genus *Trichoderma* comprise a great number of fungal strains that are the potential producer of bioactive secondary metabolites. In this study secondary metabolites from ten *Trichoderma* spp. were evaluated for their antibacterial and nematicidal potential against phytopathogenic bacteria *Ralstonia solanacearum*, *Xanthomonas compestris* and plant parasitic nematode *Meloidogyne incognita*. Five different growth media were evaluated for the production of SMs. It was shown that SMs of different *Trichoderma* spp. obtained on different growth media were different in the degree of their bioactivity. Comparison of five growth media showed that SMs produced on solid wheat and STP media gave higher antibacterial activity. SMs of *T. pseudoharzianum* (T113) obtained on solid wheat media were more effective against the studied bacteria followed by SMs from *T. asperelloides* (T136), *T. pseudoharzianum* (T129) and *T. pseudoharzianum* (T160). Scanning electron microscopy (SEM) was further conducted to observe the effect of SMs on bacterial cell morphology. As evident from the SEM, SMs produced severe morphological changes, such as rupturing of the bacterial cell walls, disintegration of cell membrane and cell content leaking out. SMs from *T. viridae* obtained on liquid STP and solid wheat media showed the highest percent of *M. incognita* juveniles (J2s) mortality and inhibition in egg hatching of *M. incognita*. The results of our study suggest that *T. pseudoharzianum* (T113) and *T. viridae* could be selected as an effective candidate for SMs source against phytopathogenic bacteria and *M. incognita* respectively.

## 1. Introduction

Both biotic and abiotic agents cause diseases in plants and pose a problem in agriculture by significantly affecting plant growth and crop yield at a global scale [1,2]. It is estimated that 10% of global food production losses are because of plant pathogens [3]. The main plant pathogens are viruses, oomycetes, fungi, bacteria and nematodes. Among the latter, there are more than 200 phytopathogenic bacterial species [4], the most important of which are *Agrobacterium*, *Pseudomonas*, *Pectobacterium*, *Xylella*, *Erwinia*, *Ralstonia*, *Dickeya* and *Xanthomonas* [5]. *Ralstonia solanacearum*, a species of the genus *Ralstonia*, is a gram-negative bacterium and is considered to be one of the most devastating plant pathogens. It has a global distribution and has a significant economic impact worldwide [6]. Another bacterium *Xathomonas campestres*, a member of genus *Xanthomonas*, is the pathogen of tomato and pepper bacterial spots, which reportedly cause 50% yield losses for tomatoes in many countries around the world [7,8]. In addition to bacterial disease, plant parasitic nematodes are also becoming a limiting factor in the successful cultivation of agricultural crops, causing an annual yield loss estimated at 8.8–14.6% of total crop production [9]. Root-knot nematodes (RKNs; *Meloidogyne* spp.) constitute major pests in agriculture worldwide, causing annual economic losses estimated at about $118 billion [10]. *M. incognita*, infecting the roots of almost all cultivated plants, is one of the most important RKN species. RKNs cause the formation of giant cells in the roots of plants, prevent the absorption of water and nutrients and promote the infection of pathogenic microorganisms [11].

Control of plant bacterial pathogens involves chemical control with streptomycin or copper and use of resistant varieties [12,13]. However, the use of resistant varieties has not always been successful, showing a negative correlation between resistance and yield [14,15]. The resistance of these varieties is often strain-specific and bacteria develop resistance to the chemicals [16,17]. In the case of RKNs, the high reproduction rate and short generation time make it difficult to control [18,19]. Chemical nematicides are usually used. Although chemical nematicides have shown good control on plant-parasitic nematodes, the public concern over the chemical nematicides is not only their toxicity but also their loss of efficiency after prolonged use [20,21]. Policies aimed at supporting environmentally friendly or sustainable agricultural practices in many countries and the increasing demand for environmental protection measures require the development of safe and effective alternatives [22]. A potential environmental and consumer-friendly opportunity to control plant diseases in crops is the use of biocontrol agents and their secondary metabolites [23].

Secondary metabolites (SMs) are small molecules that are not directly essential for growth [24,25,26]. These SMs also have an antimicrobial role against agriculturally important phytopathogens [27]. Among microorganisms, fungi are unique in SMs production, producing a vast range of SMs that are known for their capacities to secrete high levels of enzymes, antibiotics, vitamins, polysaccharides and organic acids [28]. Among fungi, the genus *Trichoderma* contains some of the most potent biocontrol agents in use today [29,30]. SMs produced by *Trichoderma* spp. can be used in medical, agricultural and industrial fields and are therefore vital to humans. These metabolites such as gliotoxin, peptaibols, gliovirin, terpenes, polyketides, and pyrones exhibit bioactivity against many phytopathogenic yeasts, filamentous fungi and bacteria [31,32,33].

The antibiotic production ability of the microorganism is generally species/isolate dependent. A given *Trichoderma* species can produce several antibiotic compounds and, in a similar way, a given antibiotic can be produced by different *Trichoderma* species [34]. Moreover, different isolates of the same species can produce different compounds [35]. Evaluating large numbers of fungal strains for antimicrobial properties in greenhouse studies on plants can be restrictively labor intensive and time-consuming. For the selection of bio-control antagonists, in vitro assays are used to demonstrate the mechanism of action of an antagonist [36]. It is the first step in the selection of effective candidates and cannot be ignored [37,38,39]. In addition, in vitro assessment to screen fungal spp. for their ability to produce bioactive compounds would facilitate the selection of the required candidate to evaluate in greater detail. Such an approach has been used successfully with other microbial organisms. For example, the efficiency of an investigation was increased for rhizobacterial strains to evaluate as control agents of *M. incognita* by first selecting those strains that produced nematicidal compounds in vitro [40]. The aim of this work was the evaluation of secondary metabolites of different *Trichoderma* spp. against plant pathogenic bacteria and parasitic nematodes. As the ability of a microorganism to produce metabolites is directly related to the type of substrate used for its growth [34], the study was also extended to investigate different growth media for the production of anti-bacterial and nematicidal SMs by *Trichoderma* spp. Moreover, a scanning electron microscopy (SEM) study was also conducted to observe the antimicrobial effect of SMs on bacterial morphology.

## 2. Materials and Methods

### 2.1. Fungal, Bacterial Cultures and Nematode Inoculum

Pre-identified and −80 °C-preserved pure cultures of ten *Trichoderma* strains including *T. pseudoharzianum* (T113), *T. koningiopsis* (T84), *T. asperelloides* (T136), *T. pseudoharzianum* (T129), *T. pseudoharzianum* (T160), *T. afroharzianum* (32233), *T. acitrinoviride* (T130), *T. longibrachiatum* (T161), *T. afroharzianum* (T52), *T. viridescens* (T196) and phytopathogenic bacteria *R. solanacearum* and *X. compestris* were obtained from the Agricultural Culture Collection of China (ACCC) and the Institute of Vegetables and Flowers, Chinese Academy of Agricultural Sciences (Beijing, China). Fungal cultures were grown on potato dextrose agar (PDA) media at 28 °C for 5–7 days [41] while bacterial cultures were grown on LB media at 28 °C for 24–48 h.

For nematode inoculum, pre-identified and pre-established egg masses of *M. incognita* were obtained from pepper plants (*Capsicum annuum* cv. Qiemen) in the greenhouses of the Institute of Vegetables and Flowers, Chinese Academy of Agricultural Sciences (Beijing, China). The egg masses were sterilized with 0.5% sodium hypochlorite (NaClO) for 1 min and washed with sterile water three times, then immersed in a Petri dish with 20 mL of sterile water at 28 °C [42]. The second-stage *M. incognita* juveniles (J2s) were collected after 24 h. Multiplication of egg masses was done by inoculating new pepper seedlings of the same cultivar with freshly hatched J2s. Both eggs and J2s were stored in 1% saline at 4 °C or immediately used in bioassays [42].

### 2.2. Extraction of Fungal Metabolites

For the extraction of secondary metabolites (SMs), five plugs of freshly prepared *Trichoderma* culture were inoculated into a 500 mL flask containing 100 mL of seed media having a composition of bacto neopeptone 10 g, maltose 40 g, yeast extract 10 g and agar 4 g/L. The flasks were incubated in the shaker at 220 RPM and 25 °C for 4 days. After 4 days of incubation, four liquid media (100 mL/flask) and one solid media (Table S1) in separate 500 mL flasks were inoculated with seed media of fungus at the rate of 5 mL per flask. The solid medium kept stationary at 25 °C while four liquid media were incubated in a shaker at 220 RPM and 25 °C for two weeks. After incubation, the flasks were poured with ethyl acetate (150 mL/flask), incubated at 25 °C at 120 RPM for 2 h and kept stationary for 30 min. The ethyl acetate layer having SMs was separated and metabolites were obtained after removing the solvent (ethyl acetate) under reduced pressure [41]. The metabolites were dried, stored at 4 °C and tested for their anti-bacterial and nematicidal activities.

### 2.3. Evaluation of Anti-Bacterial Activity

The antibacterial activity of SMs obtained on five different growth media were examined against two plant pathogenic bacteria, *R. solanacearum* and *X. compestris*, by the agar well diffusion and disk diffusion method [43]. The dried fungal metabolites were dissolved in methanol at the concentration of 150 mg/mL and used for antibacterial activity. At first, 25 mL LB medium containing 0.5 mL bacterial suspension (108 cfu) was poured in each plate and allowed to cool. Using sterilized 2 mm diameter borer, a total of seven wells were punched in each plate. Five wells each were poured with 10 µL of fungal metabolites obtaining five different growth media (STP, MMK2, SOLID, MOF and SuM), one well with 10 µL of methanol (negative control) and one with 10 µL of ampicillin 4 mg/mL (positive control). In the case of the disc diffusion method, discs were utilized instead of wells. Ten µL of fungal metabolites was poured on sterile paper discs of 5 mm in diameter and deposited on the LB medium plates inoculated with bacteria. The plates were incubated at 28 °C for 24 h. Antibacterial activity was estimated by the size (diameter in mm) of growth inhibition zones [44].

### 2.4. SEM Studies

To confirm the effect of the SMs on the in-vitro growth inhibition of the bacterial pathogen and to observe any morphological alterations occurring in the bacterial cells as a result of SMs treatment, scanning electron microscope (JSM5910, JEOL, Japan) was used. To prepare control and treated cells for morphological observations, small agar pieces were cut out from the relevant inhibition zones, fixed in 2.5% (*v*/*v*) glutaraldehyde (in phosphate buffer; pH 7.2) for 1 h at 40 °C. The pieces were then washed three times with phosphate buffer for 10 min and fixed for 2 h with 1% (*w*/*v*) osmium tetroxide (OSO_4_). This was followed by three washings (in phosphate buffer) for 10 min and dehydration in a graded ethanol series (30%, 50%, 70%, 90%, and 95%) for 15 min each. To achieve the critical point, the samples were then subjected to 100% ethanol and CO_2_. The totally dried samples were then coated with gold ion and submitted for SEM analysis [45].

### 2.5. Evaluation of Nematicidal Activity

Anti-nematode activity was demonstrated in two separate simultaneous experiments on eggs and J2s of *M. incognita*. Fungal SMs were dissolved in methanol to get the concentration of 200 mg/mL. Briefly, J2s (100) and eggs (150) of *M. incognita* contained in 100 µL of water were exposed to SMs in micro well bioassay experiments (24 well microwell plates, Sigma-Aldrich, USA). Each well was loaded with 100 µL of distilled water containing J2s (100) and eggs (150) of *M. incognita* separately, 390 µL of distilled water and 10 µl of fungal SMs (200 mg/mL). For evaluating the effect of the solvent, methanol was also used as one of the treatments. Wells containing only sterile distilled water (SDW) in place of fungal SMs served as negative control while 10 μg/mL Abamectin-loaded wells were served as positive controls. Each treatment was replicated three times and the experiment was repeated once in a completely randomized design [46]. Microwell plates were incubated in the dark at 27 °C and data on the percent of J2s mortality were recorded after incubating for 24, 48 and 72 h. J2s were defined as dead when they showed no movement and adopted straight shape despite physical stimulation with a fine needle [47]. Abbott’s formula was used for the calculation of mortality rates (M) [48] as described in Equation (1):

M = [(Mt − Mc)/(100 − Mc)] × 100(1)

where Mt presents the percent of mortality in treatment and Mc presents the percent of mortality in control.

Inhibition of egg hatching was assessed under a light microscope at seven days after treatment. Hatch inhibition (HI) was calculated by using the formula described in Equation (2):

HI = [(C − T)/C] × 100(2)

where C presents the percent of eggs hatched in the control and T presents the percent of eggs hatched in the treatment.

### 2.6. Statistical Analysis

Each treatment was replicated three times and results of the data are presented as mean value ± SD. Recorded data were analyzed by analysis of variance (ANOVA) using “Statistix” (Analytical Software, version 8.0, USA) and treatment means were compared using Fisher’s Protected LSD test at *p* = 0.05 [46].

## 3. Results

### 3.1. Antibacterial Effect of SMs from Trichoderma spp. Produced on Different Growth Media

**Figure 1:**
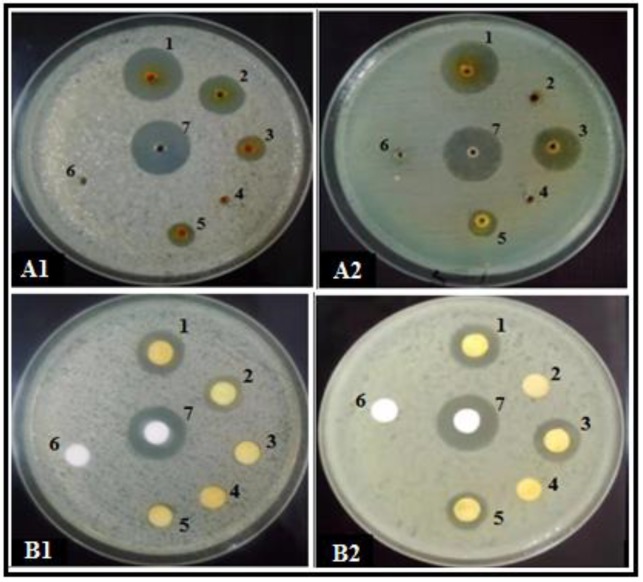
Zone of bacterial growth inhibition (mm) produced by secondary metabolites (SMs) of *T. pseudoharzianum* (T113) obtained on different growth media (1) Solid; (2) STP; (3) MOF; (4) MMK2; (5) SuM; (6) negative control, methanol; (7) positive control, ampicillin; (**A**) well diffusion method: (**A1**) *R. solanacearum*; (**A2**) *X. compestris*; (**B**) disc diffusion method: (**B1**) *R. solanacearum*; (**B2**) *X. compestris*.

**Figure 2:**
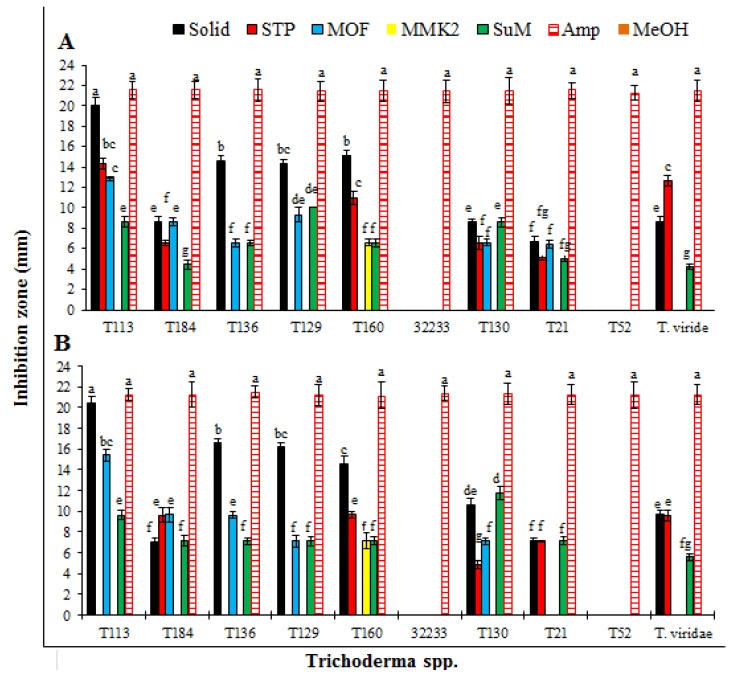
Growth inhibition (mm) of bacteria affected by SMs (obtained on different growth media) of *Trichoderma* spp. evaluated through the disc diffusion method. (**A**) Growth inhibition of *R. solanacearum*; (**B**) growth inhibition of *X. compestris*. Each value is a mean of three replicates. The bar represents the mean with standard deviation. Missing columns for some treatments indicated their 0.0 mm growth inhibition (or no activity). Treatments having similar lettering on the bar cha show no significant (*p* ≥ 0.05) difference according to Fisher’s protected LSD test.

*Trichoderma* secondary metabolites (SMs) obtained on different growth media showed significantly different results regarding bioactivity against two studied phytopathogenic bacteria. After control (ampicillin), SMs from *T. pseudoharzianum* (T113) exhibited maximum antibacterial activity compared to other strains, followed by *T. asperelloides* (T136), *T. pseudoharzianum* (T129) and *T. pseudoharzianum* (T160) (Table 1 and Table 2). The inhibition zones recorded for *T. pseudoharzianum* (T113) were 21.8 mm and 21.3 mm (solid media) for *R. solanacearum* and *X. compestris*, respectively, through the well diffusion method (Figure 1: A1,2). Similar results were seen when bioactivity was checked through the disc diffusion method, where SMs of *T. pseudoharzianum* (T113) produced an inhibition zone of 20.0 and 20.4 mm against *R. solanacearum* and *X. compestris* through the disc diffusion method (Figure 1: B1,2). The SMs of *T. asperelloides* (T136), *T. pseudoharzianum* (T129) and *T. pseudoharzianum* (T160) showed inhibition zones of 15.1, 14.2, 15.3 mm against *R. solanacearum* and 14.8, 15.2, 15.5 mm against *X. compestris* through the well diffusion method (Table 1 and Table 2). The disc diffusion method of these three strains also gave similar activity (Figure 2). Metabolites from other strains either exhibited very little bioactivity or showed no activity. The positive control ampicillin gave the highest value for the zone of inhibition while the negative control methanol did not affect the bacterial growth.

### 3.2. SEM Observations of the Bacterial Cells

**Figure 3:**
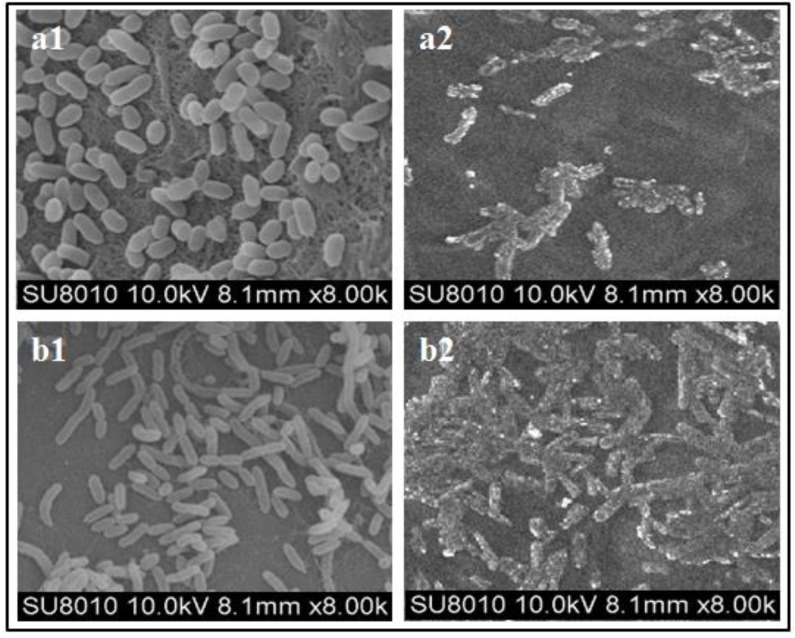
SEM micrograph of (**a1**) untreated normal *R. solanacearum* cells; (**a2**) treated *R. solanacearum* cells with SMs of *T. pseudoharzianum* (T113) obtained solid media; (**b1**) untreated normal *X. compestris* cells; (**b2**) treated *X. compestris* cells with *T. pseudoharzianum* (T113).

In order to observe the effect of SMs on morphological changes of bacterial cells, the most effective SMs (*T. pseudoharzianum* T113) treated bacterial cells and untreated bacterial cells were subjected to SEM study. SEM micrographs of treated bacteria and without treatment are illustrated in Figure 3. Cell wall degradation and morphology revealed that the bacterium was exposed to treatment and was absolutely different from the control (untreated). The characteristic morphology of the control group displayed a uniform rod-shaped surface of *R. solanacearum* (Figure 3a1) and *X. compestris* (Figure 3b1) while SM-treated cells showed enormous morphological alterations both in *R. solanacearum* (Figure 3a2) and *X. compestris* (Figure 3b2). Under the influence of SMs the bacterial cells seem misshaped, their cell wall degraded, the cell membranes disintegrated and cell contents leaked out.

### 3.3. Nematicidal Effect of SMs of Trichoderma spp. against Meloidogyne Incognita

**Figure 4:**
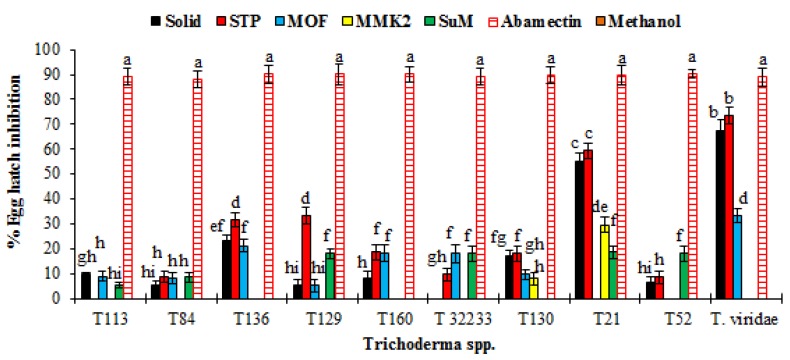
Effect of SMs of *Trichoderma* spp. obtained on different growth media on egg hatching of *M. incognita*. Values are the mean of three replicates. The bar represents the mean with standard deviation. Missing columns for some treatments indicated their 0.0% egg hatch inhibition (or no activity). Treatments having similar lettering on the bar chart show no significant (*p* ≥ 0.05) difference according to Fisher’s protected LSD test.

**Figure 5:**
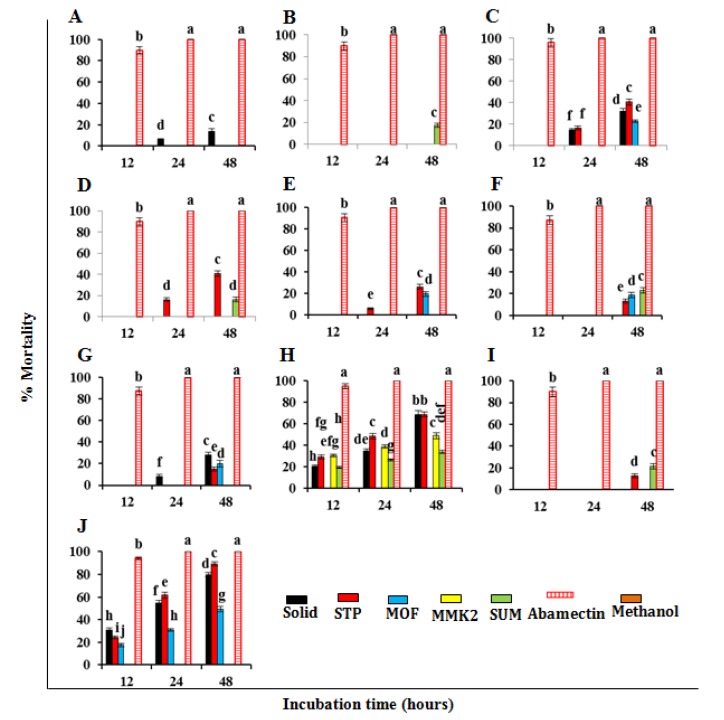
Effect of SMs of *Trichoderma* spp. obtained on different growth media on *M. incognita* juveniles (J2s) mortality of *M. incognita*. Values are the mean of three replicates. (**A**) *T. pseudoharzianum* (T113); (**B**) *T. koningiopsis* (T84); (**C**) *T. asperelloides* (T136); (**D**) *T. pseudoharzianum* (T129); (**E**) *T. pseudoharzianum* (T160); (**F**) *T. afroharzianum* (32233); (**G**) *T. acitrinoviride* (T130); (**H**) *T. hamatum* (T21); (**I**) *T. afroharzianum* (T52); (**J**) *T. viridae*. The bar represents the mean with standard deviation. Missing columns for some treatments indicated their 0.0% mortality (or no activity). Treatments having similar lettering on the bar chart show no significant (*p* ≥ 0.05) difference according to Fisher’s protected LSD test.

Results of nematicidal activity of SMs revealed that only SMs of *T. viridae* and *T. hamatum* (T21) effectively inhibited the hatching of *M. incognita* eggs. Among *Trichoderma* spp. the highest egg hatch inhibition (71.6%) was achieved by SMs of *T. viridae* obtained on STP media followed by 67.3% of solid media (Figure 4). The second effective egg hatch inhibition of 59.2% and 54.7% was recorded with SMs of *T. hamatum* (T21) obtained on STP and solid media, respectively. Results of remaining tested *Trichoderma* spp. were not effective, showing very little or no effect (Figure 4). A similar trend was observed in the case of percent of juvenile mortality. After positive control the highest percent juvenile mortality was achieved by *T. viridae* on STP and solid media, respectively (Figure 5), followed by *T. hamatum* (T21). SMs from remaining *Trichoderma* spp. had little effect or were not effective (Figure 5).

### 3.4. Morphological Variations of M. incognita J2 under the Influence of SMs

**Figure 6:**
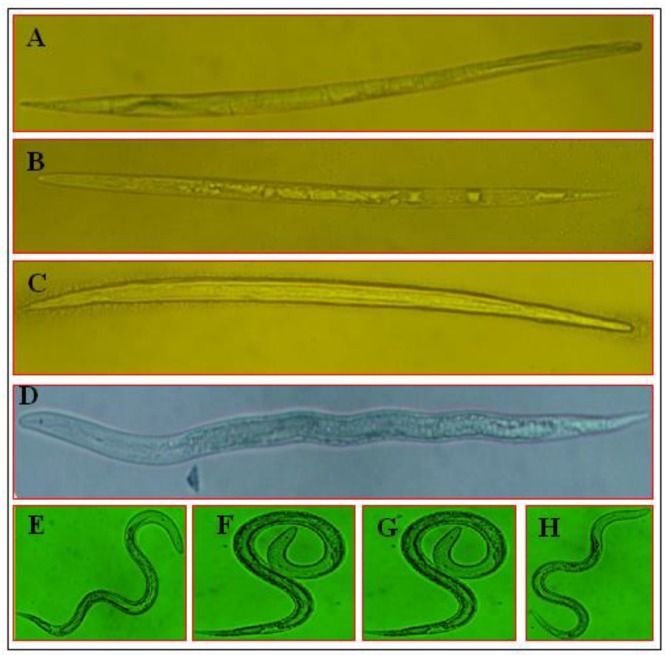
Morphological variations in *M. incognita* J2s treated with SMs of *T. viridae* obtained on different growth media. (**A**) Abamectin (positive control); (**B**) STP; (**C**) Solid; (**D**) MOF; (**E**) MMK2; (**F**) SuM; (**G**) methanol; (**H**) water (negative control). Bar = 300 μm.

For the observation of morphological variations of *M. incognita* J2 under the influence of SMs, the results of *T. viridae* SMs (most effective) are presented in Figure 6. Microscopic observation indicated that the most effective SMs (*T. viridae* on STP and solid media) probably infected and destroyed internal organs, resulting in straightened and stiffened body shape (Figure 6B,C); similar observations were also recorded for positive control (Abamectin) (Figure 6A). In comparison, the exposure to *T. viridae* SMs obtained on MOF media showing lower effectiveness caused relatively less straightness and stiffness of the body (Figure 6D) while treatment of J2s with MMK2, SuM media, methanol and negative control, having no effect on J2s mortality, did not induce such morphological changes and the nematodes possessed a natural posture (Figure 6E–H).

## 4. Discussion

Plant pathogens cause catastrophic loss in different crops and are becoming increasingly serious threats to global food security. Huge genetic diversity, survival capacity in various environments and wide host range make them difficult to control. Therefore, an integrated approach combining host plant resistance and cultural and biological control measures seems effective. Although excellent attempts have been made in the management of plant pathogens, still there is great opportunity to contribute to this problem by finding a stable solution. Different agronomical, biochemical, chemical, cultural and biotechnological approaches have been utilized in addressing the problem of plant pathogens with different levels of success. Management of plant pathogens through bioactive secondary metabolites of bio control agents have been reported as a potential environmental and consumer-friendly approach [23]. Scientists diverted towards fungi because of their diversity in metabolites for the source of natural products that can be used effectively for the management of pathogenic microorganisms. Fungi are known to play an important role in biotechnology applications and their SMs have recently been used as effective biological pesticides [49]. Most of the antimicrobial metabolites are mainly derived from soil-borne fungi and these microorganisms are less explored [50]. Analysis of antimicrobial activity of these metabolites against pathogens can be employed as a simple and efficient screening tool and could be used to isolate potentially useful strains and to further identify their bioactive agents [51]. This work investigated the antimicrobial potential of *Trichoderma* SMs obtained on different growth media against plant pathogenic bacteria and parasitic nematode.

The results of our study indicated that SMs secreted by different *Trichoderma* spp. have the potential to inhibit the growth of two economically important plant pathogenic bacteria, *R. solanacearum* and *X. compestris*. Different *Trichoderma* species have also been reported previously for the suppression of phytopathogenic bacteria [52]. Among other mechanisms adopted by *Trichoderma* spp., the production of various bioactive SMs is of great importance [53]. Results showed that all of the ten *Trichoderma* strains are not producing bioactive secondary metabolites against *R. solanacearum* and *X. compestris*. SMs of some strains showed higher activity, some showed lower activity, while others did not affect the growth of bacteria at all. Significantly higher anti-bacterial activity was achieved by the application of SMs obtained from strains belonging to *T. pseudoharzianum* sp. and *T. asperelloides* sp. This is because the production of bioactive SMs depends on strains and species of the fungal agent. It is reported that different *Trichoderma* isolates are not effective equally against pathogens in in vivo and in vitro conditions [54]. During our study, significant zones of inhibition were observed. These growth-free zones are possibly because of the presence of different anti-bacterial compounds present in *Trichoderma* metabolites. Various anti-bacterial compounds were reported previously from *Trichoderma* SMs. For example, *T. harzianum* controlled *Clavibacter michiganensis* subsp. *michiganensis* by producing Lysosime [55]. *T. harzianum* T23 prevented growth of *Erwinia amylovora* and *C. michiganensis* in vitro by producing viridiofungin A (VFA) [56]. Different peptaibols such as Trichokonin VI, VII and AVIII were identified in *Trichoderma* spp., which are the cause of the suppression of bacterial growth [54].

Bio-active compounds damage bacterial cells by different mechanisms. Alkaloids may inhibit important enzymes or act as DNA-intercalating agents [57]. Some flavonoids coagulate soluble bacterial cell proteins, including important enzymes, by making complexes with them [58]. Others are responsible for membrane disruption [59] and inhibition of cell wall and nucleic acid synthesis [60,61]. Lysosime affects bacterial cell walls and the cell membrane, leading to membrane disruption and the release of intracellular contents and consequent bacterial cell death [62]. These mechanisms of action are consistent with our SEM studies. The micrographs show clear morphological changes that occurred as a result of treatment of the bacterial cells with SMs. The treated cells, in comparison to untreated cells, seemed to be severely damaged. Their cell walls were broken, their cell membranes were disrupted, and cell contents leaked out.

The ability to produce antibiotics is dependent on the microorganism, environment (pH and temperature) and substrate. Presence of proper medium to produce bioactive compounds is necessary because it satisfies the needs of metabolite production [63]. Our results indicated that different growth media have significant roles in the production of antibacterial and nematicidal SMs. SMs obtained on different growth media have different effects in term of bacterial growth inhibition and nematicidal activity. The SMs having the highest antibacterial activity were produced by *Trichoderma* spp. on wheat solid media. Different scientists have also evaluated different growth media for optimizing the production of antimicrobial SMs by the fungal spp. It was reported that fungal extracts, which are prepared by using different growth media, exhibited different inhibition zones against many phytopathogenic bacteria [64,65]. Similarly, another study evaluation of different growth media revealed that growth medium’s nutrient composition highly affects SMs [66]. Anti-bacterial activity can be improved by nutrient composition of media [67]. It has been recommended that that particular growth medium may result in producing a greater concentration of many bioactive compounds that may improve the activity [68].

Among all the studied *Trichoderma* spp., only SMs produced by *T. virens* showed significantly strong nematicidal activity causing the highest egg hatch inhibition and juvenile death. The production and bioactivity of nematicidal SMs depends on growth media, such as those metabolites obtained on STP and wheat solid media that showed higher activity. These results are in agreement with previous reports, such as when filtrates from *T. virens* were evaluated against *M. incognita* and it was found that the medium used for culturing the microbes affected the production of bioactive compounds [69]. In another study it was demonstrated that the bioactivity of cultural filtrates from *T. virens* against *M. incognita* depends on growth media [70], and potential biocontrol of *T. virens* against *M. incognita* has been achieved [71]. The bioactivity of SMs against *M. incognita* could be attributed to the nematicidal compounds. Several nematicidal compounds have been isolated from *Trichoderma* spp., such as acetic acid that was identified as the nematicidal principle in the culture filtrate of *T. longibrachiatum* [72], and Glio-toxin that has been isolated from a large number of fungi including a strain of *T. virens* which showed nematicidal activity [73]. A peptide cyclosporin A possessing nematicidal activity against *M. incognita* was obtained from *T. polysporum* [74]. Mortality of *M. incognita* J2s can also be explained on the basis of disintegration of nematode tissues. In previous studies, it was reported that application of SMs against *M. incognita* J2s resulted in the disintegration of internal tissue of the nematodes that eventually caused death [75,76].

The crude extracts of *Trichoderma* may contain a variety of bioactive SMs that can be used for the management of *R. solanacearum* and *M. incognita*. Our findings help in the selection of the right candidates in order to study them in greater depth for the identification of bioactive molecules of industrial interest or in commercial formulations of products for biological control of *R. solanacearum* and *M. incognita*. In the context of facing the increasing demand of agriculture for ecologically compatible alternatives for the management of plant diseases, we showed the potential of *Trichoderma* spp. for the production of broad-spectrum bioactive metabolites against plant pathogens.

## 5. Conclusions

This study concludes that SMs from *Trichoderma* spp. significantly inhibited the bacterial growth and damaged the bacterial cells of *R. solanacearum*. They caused significant J2s mortality and inhibition in egg hatching of *M. incognita*. The bioactivity of SMs from *Trichoderma* spp. could increase their potential application in the biological control of plant diseases. *Trichoderma* spp. can be used as a potential source for the isolation of bioactive SMs against *R. solanacearum* and *M. incognita*. Utilization of these secondary metabolites could act as an effective, non-chemical and eco-friendly disease management tool against *R. solanacearum*, *M. incognita* and other plant pathogens.

## Figures and Tables

**Table 1:** Growth inhibition (mm) of *R. solanacearum* affected by SMs (obtained on different growth media) of *Trichoderma* spp. evaluated through the well diffusion method.

*Trichoderma* spp.	Growth Media					Control	
	Solid	STP	MOF	MMK2	SuM	Ampicillin	Methanol
*T. pseudoharzianum* (T113)	21.8 ± 0.4^a^	14.3 ± 0.4^c^	12.2 ± 0.8^d^	0.0^s^	9.0 ± 0.9^hij^	22.3 ± 0.5^a^	0.0^s^
*T. koningiopsis* (T84)	9.7 ± 0.5^gh^	7.1 ± 0.3^m^	8.4 ± 0.2^ijk^	0.0^s^	4.2 ± 0.1^qr^	22.0 ± 0.5^a^	0.0^s^
*T. asperelloides* (T136)	15.1 ± 0.2^bc^	0.0^s^	7.0 ± 0.4^mn^	0.0^s^	7.8 ± 0.1^klm^	22.2 ± 0.3^a^	0.0^s^
*T. pseudoharzianum* (T129)	14.2 ± 0.5^c^	0.0^s^	10.5 ± 0.1^fg^	0.0^s^	9.0 ± 0.1^hij^	22.4 ± 0.2^a^	0.0^s^
*T. pseudoharzianum* (T160)	15.3 ± 0.7^b^	11.2 ± 0.3^ef^	0.0^s^	6.1 ± 0.1^no^	6.0 ± 0.1^o^	21.6 ± 0.2^a^	0.0^s^
*T. afroharzianum* (32233)	0.0^s^	0.0^s^	0.0^s^	0.0^s^	0.0^s^	21.8 ± 0.3^a^	0.0^s^
*T. acitrinoviride* (T130)	8.2 ± 0.1^jkl^	5.2 ± 0.2^op^	7.0 ± 0.0^mn^	0.0^s^	7.3 ± 0.5^lm^	22.3 ± 0.2^a^	0.0^s^
*T. hamatum* (T21)	5.2 ± 0.2^op^	4.5 ± 0.1^pq^	6.1 ± 0.2^no^	0.0^s^	3.3 ± 0.1^r^	22.5 ± 0.1^a^	0.0^s^
*T. afroharzianum* (T52)	0.0^s^	0.0^s^	0.0^s^	0.0^s^	0.0^s^	21.7 ± 0.2^a^	0.0^s^
*T. viridae*	9.2 ± 0.1^hi^	12.1 ± 0.1^de^	0.0^s^	0.0^s^	4.4 ± 0.1^pq^	22.2 ± 0.1^a^	0.0^s^

Values are the mean of three replicates ± SD (Standard deviation). Those mean values having the same lettering in a row or column are statistically not significant (*p* ≤ 0.05) from each other (Fisher’s protected LSD test). LSD = 0.92 in Fisher’s protected LSD test.

**Table 2:** Growth inhibition (mm) of *X. compestris* affected by SMs (obtained on different growth media) of *Trichoderma* spp. evaluated through the well diffusion method.

*Trichoderma* spp.	Growth Media					Control	
	Solid	STP	MOF	MMK2	SuM	Ampicillin	Methanol
*T. pseudoharzianum* (T113)	21.3 ± 0.1^b^	0.0^n^	16.8 ± 0.2^c^	0.0^n^	10.3 ± 0.5^e^	21.9 ± 0.5^ab^	0.0^n^
*T. koningiopsis* (T84)	7.8 ± 0.3^gh^	7.1 ± 0.1^ij^	9.5 ± 0.1^f^	0.0^n^	4.5 ± 0.2^m^	22.2 ± 0.1^a^	0.0^n^
*T. asperelloides* (T136)	14.8 ± 0.3^d^	0.0^n^	7.1 ± 0.1^ij^	0.0^n^	6.2 ± 0.4^k^	21.7 ± 0.4^ab^	0.0^n^
*T. pseudoharzianum* (T129)	15.2 ± 0.2^d^	0.0^n^	9.2 ± 0.1^f^	0.0^n^	6.4 ± 0.1^k^	21.7 ± 0.2^ab^	0.0^n^
*T. pseudoharzianum* (T160)	15.5 ± 0.2^d^	9.7 ± 0.1^ef^	0.0^n^	7.4 ± 0.2^hi^	5.2 ± 0.2^lm^	22.3 ± 0.1^a^	0.0^n^
*T. afroharzianum* (32233)	0.0^n^	0.0^n^	0.0^n^	0.0^n^	0.0^n^	21.8 ± 0.4^ab^	0.0^n^
*T. acitrinoviride* (T130)	9.5 ± 0.2^f^	4.9 ± 0.1^lm^	6.5 ± 0.2^jk^	0.0^n^	9.3 ± 0.1^f^	22.0 ± 0.4^a^	0.0^n^
*T. hamatum* (T21)	5.2 ± 0.1^lm^	4.8 ± 0.1^m^	5.5 ± 0.1^l^	0.0^n^	4.9 ± 0.3^lm^	22.2± 0.1^a^	0.0^n^
*T. afroharzianum* (T52)	0.0^n^	0.0^n^	0.0^n^	0.0^n^	0.0^n^	21.8 ± 0.4^ab^	0.0^n^
*T. viridae*	8.4 ± 0.2^g^	9.5 ± 0.1^f^	0.0^n^	0.0^n^	4.9 ± 0.3^lm^	22.1 ± 0.2^a^	0.0^n^

Values are the mean of three replicates ± SD (Standard deviation). Those mean values having the same lettering in a row or column are statistically not significant (*p* ≤ 0.05) from each other (Fisher’s protected LSD test). LSD = 0.67 in Fisher’s protected LSD test.

## Supplementary Materials

The following are available online at https://www.mdpi.com/2076-2607/8/3/401/s1. Table S1. Composition of five growth media used for the production of secondary metabolites.
