# Supplementary material for: Bioactive Secondary Metabolites from Trichoderma spp. against Phytopathogenic Bacteria and Root-Knot Nematode
Source: Microorganisms. 2020 Mar 13;8(3):401. doi: 10.3390/microorganisms8030401 (PMC7143365; doi:10.3390/microorganisms8030401)
Supplement: Supplementary file 1 [file microorganisms-08-00401-s001.pdf]

Supplementary Table

Table S1. Composition of five growth media used for the production of secondary metabolites.

Ingredients	Amount (g)/L
**(1) MMK2**	
Mannitol	40.0
Yeast extract	5.0
Mrashuge & Skoog salts	4.3
**(2) STP**	
Sucrose	75.0
Tomato paste	10.0
Malt extract	5.0
$(NH_4)_2 SO_4$	1.0
Soy flour	1.0
KH_2PO_4	9.0
**(3) MOF**	
Mannitol	75.0
Oat flour	15.0
Yeast Extract	5.0
L-glutamic acid	4.0
MES	16.2
**(4) Supermalt (SuM)**	
Malt extract	50.0
Yeast extract	10.0
$FeSO_4$. $7H_2O$	20.0
$ZnSO_4$. $7H_2O$	7.0
**(5) Wheat Solid***	
Wheat	30 g

*For the preparation of Wheat solid media, 50 ml base liquid media (Yeast autolsate 2.0 g, Sodium tartrate 10.0 g, KH_2PO_4 1.0 g, $MgSO_4.7H_2O$ 1.0 g, $FeSO_4.7H_2O$ 1.0 g/L) was poured on 30 g wheat in 500 ml flask.
